# Retail pharmacy prescription medicines’ availability, prices and affordability in Eswatini

**DOI:** 10.4102/phcfm.v13i1.2986

**Published:** 2021-09-15

**Authors:** Garikai Shambira, Fatima Suleman

**Affiliations:** 1 Discipline of Pharmaceutical Sciences, School of Health Sciences, University of KwaZulu-Natal, Durban, South Africa

**Keywords:** Eswatini, comparison, South Africa, retail medicines’ prices, affordability, availability

## Abstract

**Background:**

Limited availability of medicines in public facilities and unaffordable prices in the private sector act as barriers to medicines’ access. Patients in Eswatini may be forced to buy medicine from the private sector resulting from chronic medicines’ shortages in public health facilities. The extent to which they can afford to do so is unknown.

**Aim:**

To determine the availability, price and affordability of medicines in the retail pharmacies in Eswatini, and to compare the results regionally and internationally.

**Setting:**

Retail pharmacy sector in the four administrative regions of Eswatini.

**Methods:**

Data on availability, price and affordability to patients for 50 medicines in the originator brand (OB) and the lowest priced generic (LPG) equivalent, were collated using the standardised World Health Organization/Health Action International methodology from 32 retail pharmacies in the four regions of Eswatini. Prices were then compared with selected countries.

**Results:**

The overall mean availability of all medicines in selected retail pharmacies was 38.5%; standard deviation [s.d.] = 20.4% for OBs and 80.9%; s.d. = 19.0% for LPGs. The overall median price ratio (MPR) in the surveyed pharmacies was 18.61 for the OBs and 4.67 for LPGs. Most standard treatments with LPGs cost less than a day’s wages whilst for OBs cost more than a day’s wages. The differences between Eswatini and South African prices were statistically significant.

**Conclusion:**

Drug pricing policies and price monitoring tools are needed for the whole pharmaceutical chain in Eswatini to monitor availability, affordability and accessibility of medicines to the general populace.

## Introduction

Although medicines are crucial in the healthcare system, they are generally not affordable to people globally. It is well documented in literature that prohibitive pricing is one of the major barriers to essential medicines’ access.^1,2,3^ There is significant underuse of costly medicines in populations that do not have any medical insurance, and thus, even the smallest price changes in drugs will impact adherence significantly amongst the poor.^4^

More than 30% of the world population does not have reliable access to essential medicines.^5^ In some of the poorest countries in Asia and Africa, the proportion is as high as 50%. Unavailability and/or low availability of essential medicines in the public health outlets may become a major barrier to medicines’ access, especially when coupled with high unaffordable prices in the private sector.^6^ It is thus imperative that prices in the private sector be affordable to ensure equitable access.^7,8^

Medicines’ expenditure constitutes between 20% and 60% of health expenditure in low- and middle-income nations compared to just 18% in the European nations. In developing countries, only a mere 10% have health insurance and the rest buy medication through out-of-pocket payments, which makes medication the biggest family expenditure item after food.^9^

Different policy options are available to governments to regulate pharmaceutical pricing such as free pricing, price-regulated options, price differentiation, price competition and discounts, and tendering procedures.^10^ Different schemes are used globally to regulate drug prices; for example, France and Italy utilise drug price controls to directly manage the drug prices. In Germany and Japan, prices are indirectly controlled via reimbursement under social insurance schemes. The National Pharmaceutical Price Authority in the United Kingdom (UK) monitors price by regulating the profits that companies make on branded prescription medicines’ sales.^11^ It is widely believed that the drug prices are generally higher in countries with less stringent price regulation (like the UK) or no regulation at all (the United States [US]) as compared to countries with strict price regulation.^12^

The market share of originator brands (OBs) reduces significantly after the introduction of generics after the expiration of patent protection, and the competition between the generic options lowers the prices of the branded products further.^12^ Free-pricing systems may lower the medicines’ prices when optimum conditions are created, and as much as regulation in price-controlled systems may reduce the prices of both generics and brands, it may become a barrier to incentives to lowering prices below the listed ones.^10^

Studies in the US in price variability with other commodities besides pharmaceuticals found that the poorer individuals usually find themselves paying more for similar goods and services as compared to their richer counterparts.^13^ The grocery stores in poorer locations are usually smaller and more expensive than in the wealthier suburbs, mainly because of the existence of large chain stores in the more affluent areas and mainly independents in the poorer areas.^13,14^ Not much is known about price regulation or affordability in low- and middle-income countries, especially across the African region.

Eswatini is classified as a lower-middle-income country with the majority of the population living below the upper poverty line.^15^ Although Mhlanga et al.^16^ recommended implementation of a pharmaceutical pricing policy in 2016, there is still no price regulation of pharmaceuticals in Eswatini.^17^ Mhlanga et al.^16^ highlighted the importance of reliable evidence on medicines’ prices in ascertaining the type of challenges in a system, before deciding on solutions to ensure availability of essential medicines at the lowest possible price to the consumer.

Prices for prescription medicines are a significant obstacle to appropriate medicine use.^18^ It is not easy to find reliable medicines’ prices’ information in developing countries,^19^ including Eswatini. The World Health Organization/Health Action International (WHO/HAI) has set a benchmark of 80% medicine availability as high to ensure the supply of essential medicines.^20^ Although generic medicines are way cheaper than the OBs, they are still relatively unaffordable in many parts of developing countries.^21^

The Medicines Regulatory Unit in the Ministry of Health is currently responsible for medicines’ registration; however, a medicines’ regulatory authority and pharmacy council are still to be established in Eswatini.^22^ All prescription medicines (defined as any medicine on a valid doctor’s prescription) attract value added tax (VAT) at 0%, and where there is no prescription, VAT is levied at 15%.^17^ Cross-national differences in pharmaceutical prices are of great importance as they help governments to come up with appropriate domestic pricing policies.^23^

South Africa, as Eswatini’s neighbour, is considered as an upper-middle-income country and has introduced price controls for medicines. The single exit price (SEP) mechanism lists the price that a medicine can be sold by a manufacturer to an end dispenser. The *South African Medicines and Related Substances Act* (as amended) regulates the maximum additional dispensing fee that can be charged by people licensed to dispense and retail pharmacists, based on a tier structure directly tied to the SEP. All retail medicines attract a 15% VAT.^24^ The Act has a provision that prohibits the use of bonuses, rebates or any incentives in the supply of medicines, to avoid undermining the SEP.^25^

The aim of this study was to investigate the availability, affordability and prices that people pay for medicines in different parts of Eswatini. In addition, this study sought to ascertain how the medicines’ prices in Eswatini were compared to the prices of the same medicines in South Africa and internationally.

## Research methods and design

### Study design

A quantitative study using a cross-sectional descriptive design was employed.

### Setting

**FIGURE 1:**
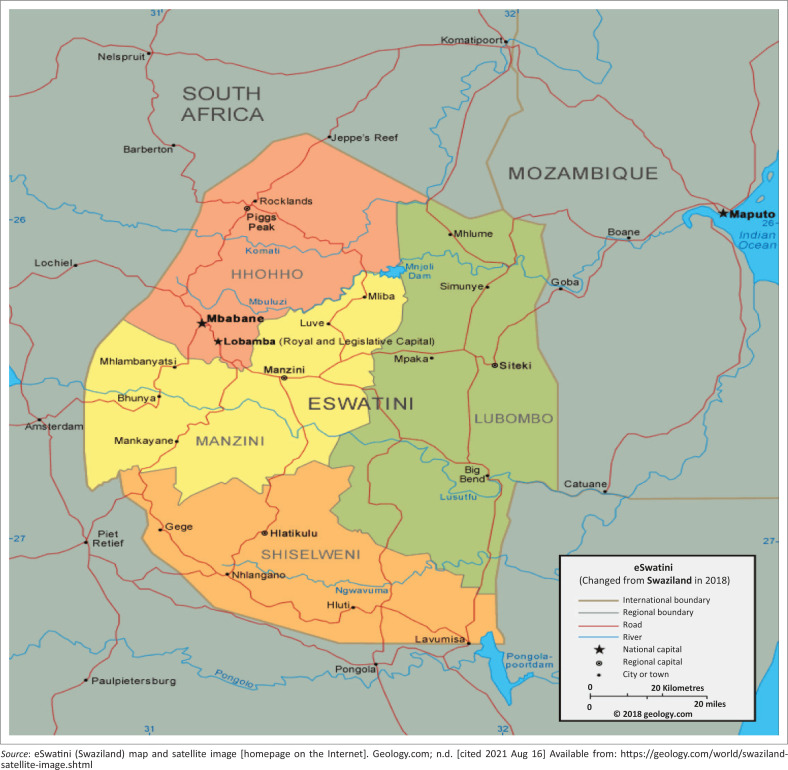
Eswatini (formerly Swaziland) map.

Eswatini is a very small country, at just over 17 000 km² and a population of just over a million people and a population density of 66.1% people/m². It is located in southern Africa and is bordered by South Africa and Mozambique (see Figure 1). The country is divided into four regions: Hhohho (north–west: 28.5% of the population), Manzini (central: 30.5% of the population), Lubombo (east: 21% of the population) and Shiselweni (south: 20.5% of the population).^27^

The urban population in Eswatini is 24.2%, and the unemployment rate was estimated at 23.4% in 2020.^27^ Consultation fee is levied at $1.41 in the public sector, and medicines are free.^16^ The lowest paid government worker in Eswatini earns a daily wage of $8.86 ($1.00 = Swazi lilangeni [SZL] 17.103 according to the exchange rate as at 15 June 2020).^28^ Less than 20% afford medical insurance, and patients in Eswatini may be forced to buy medicines from the private sector because of the chronic medicines’ shortages in Eswatini public health facilities.^16^

Pricing surveys have been used to determine the extent of price differentiation and affordability in countries. Thus, for Eswatini, the WHO/HAI medicine price survey was used to identify medicine pricing and affordability in the country in order to provide policymakers and all stakeholders with evidence to draft policies that may improve availability, affordability and accessibility of medicines.^6,20^

### Study population and sampling strategy

A list of pharmacies was obtained from the office of the Deputy Director of Pharmaceutical Services in the Ministry of Health of the 60 registered retail pharmacies in Eswatini at the time of the study, with 14 geographically situated in Hhohho, 30 in Manzini, 10 in Lubombo and six in the Shisweleni region. Stratified sampling was used to group the pharmacies. The primary criterion was the region of location for the pharmacy.

A total number of 32 pharmacies were included in the study. Random disproportionate stratified sampling was used to ensure that the sample was representative of the Eswatini retail pharmacy population. The sample comprised 12 pharmacies from Manzini, eight from Hhohho, six from Lubombo and six from Shiselweni. Simple random sampling was used to select the pharmacies to use in each region. To analyse the effect of the size of the city/town on prescription prices, pharmacies were categorised as from either city or town/rural area. Categorisation of pharmacy size was based on the population of the location:

- City population ≥ 50 000
- Town/rural population < 50 000

### Data collection

The survey included a total of 50 medicines (Appendix Table 1-A1). The list comprised the WHO/HAI Global Core List of 14 medicines and 36 supplementary lists based on the disease burden and local relevance in Eswatini. Expert advice was sought from pharmacists, medical doctors, academicians and Ministry of Health Professionals on the relevance of the selected medicines. For analysis, medicines were stratified by use (i.e. anti-infectives [AI] and non-communicable disease [NCD] medicines) and also on whether they were on the essential medicines list (EML) or not.

The managers and/or responsible pharmacists of the selected facilities were contacted via email and telephonically explaining the objectives of the study and the information that would be collected from their pharmacies. The responsible were informed that they were not obliged to participate in the study and that all identity information would be kept confidential.

Data collection took place between 15 June 2020 and 13 August 2020. Data on price, availability and affordability to patient were collected for two products, namely the OB and the LPG using the standardised WHO/HAI methodology.^29^ The following specific data were collected for 50 essential medicines as per the WHO listing (14 from the Global Core List and 36 selected based on local relevance),^30^ using the WHO/HAI workbook,^31^ during visits to ‘retail pharmacies’:

- The name of pharmacy, administrative region where located, and whether it was in a city or town/rural area.
- Brand/product name and manufacturer of the LPG found at the site.
- Availability of the OB and the LPG.
- Pack size and price of the pack found for the OB and the LPG.
- Any other comments regarding a given product.

During the retail pharmacy visits, data were recorded on hard copy medicine prices’ data collection forms. The data collector made sure the data collection forms were complete and legible before leaving an outlet.

As per the standardised methodology, data collection forms were reviewed every day after completion of the fieldwork to ensure data quality. The data were then entered from the hard copy forms into the electronic survey workbook, and the double-entry programme was run, and any mistakes were corrected. Any questionable data identified after running data checker were investigated and corrected.

### Data analysis

Data were entered in the pre-programmed WHO/HAI Microsoft Excel Workbook.^31^ The workbook automatically generated analysis of the data entered once complete, giving summary tables of percentage availability and median price ratios (MPRs).^31^ Median local prices were expressed as ratios to international reference prices (IRPs), using the formula:

$$\text{Medicine price ratio (MPR)}=\frac{\text{median local unit price}}{\text{International reference unit price}}$$ [Eqn 1]

Management Sciences for Health (MSH)’s 2015 prices were used as the default IRPs.^32^ The IRPs used are prices offered to international not-for-profit agencies for purchase of generics.

The availability of medicines was determined as a percentage of outlets where medicine was found on the day of data collection. Mean availability of the 50 medicines was also reported. The differences in average percentage availability of OBs and LPGs determined if there was any variance in the availability of the two product types. To describe availability, the following ranges were used as reference:^19^

- < 30% extremely low
- 30% – 49% low
- 50% – 80% fairly high
- > 80% high.

The MPR pharmacy prices for each drug were compared across region categories using analysis of variance (ANOVA). The MPR pharmacy prices for each drug were compared between the two categories of location size using *t*-test. The lowest and maximum prices in Eswatini were compared to the lowest and highest permissible retail prices in South Africa (based on current dispensing fee guide in South Africa at the time),^33,34^ and the differences were expressed as percentage price difference for similar products and were analysed using the *t*-test for statistical significance.

Affordability was calculated as the number of wage days that the lowest paid government worker needed to spend to pay for treatment and was based on the median local price of a medicine prescribed at a standard dose. All analysis was carried out using Statistical Package for Social Sciences (SPSS) version 27 (University of KwaZulu-Natal, School of Health Sciences).

## Results

### Distribution of pharmacies

A total number of 32 pharmacies were included in the study. Twelve pharmacies were drawn from Manzini region, eight pharmacies from Hhohho region, six pharmacies from Lubombo region and six pharmacies from Shiselweni region. Of the 32 surveyed pharmacies, 13 of them were located in cities and 19 in towns and rural areas.

### Medicines’ availability on the day of data collection

The overall mean availability of all medicines in the surveyed retail pharmacies was 38.5%; standard deviation [s.d.] = 20.4% for OBs and 80.9%; s.d. = 19.0% for LPGs. Table 1 highlights the mean availability of the different classes of medicines in the different regions.

**TABLE 1:** Mean availability of selected product groups in the different regions.

All	Mean availability Manzini (%)	Mean availability Hhohho (%)	Mean availability Shiselweni (%)	Mean availability Lubombo (%)	Overall mean availability Eswatini (%)	*N*
LPGs	83.3	82.4	79.6	76.2	80.9	-
OBs	42.3	56.1	28.5	23.6	38.6	-
**Medicine type**						
Anti-infectives	-	-	-	-	-	8
LPGs	85.7	87.5	88.9	71.4	86.7	-
OBs	37.5	39.6	23.3	13.3	30.7	-
NCD medicines	-	-	-	-	-	42
LPGs	82.9	81.1	83.3	76.4	82.3	-
OBs	41.9	21.4	29.2	21.4	38.5	-
**Essential medicines**						
On EML	-	-	-	-	-	38
LPGs	86.7	88.9	84.2	80.2	85.1	-
OBs	37.6	57.2	18.4	25.6	38.3	-
Not on EML	-	-	-	-	-	12
LPGs	72.9	69.8	65.3	62.5	69.0	
OBs	40.2	49.0	26.7	19.4	40.0	
**Sample list**						
Global core	-	-	-	-	-	14
LPGs	89.3	91.1	88.1	88.1	89.2	-
OBs	41.7	48.8	35.0	30.0	40.0	-
Supplementary	-	-	-	-	-	36
LPGs	81.0	78.9	76.2	71.4	77.7	-
OBs	42.6	58.8	26.2	22.0	38.0	-

OB, originator brand; LPG, lowest priced generic equivalent; NCDs, non-communicable diseases; EML, essential medicines list.

*n* = 50.

Although availability data only refer to the day of data collection, the availability of OBs was generally low in all the surveyed pharmacies with only Hhohho region recording more than 50% availability (56.1%), whilst Manzini had 42.3% availability, Shiselweni 28.5% and then Lubombo with 23.6% availability. Analysis of variance results showed there were significant differences between the regions in terms of OB availability (*p* < 0.001). A post hoc test (Bonferroni’s correction) showed that Hhohho had significantly higher OB availability than Manzini, Shiselweni and Lubombo regions (*p* < 0.001). The difference between Manzini and Lubombo (*p* = 0.170), Manzini and Shiselweni (*p* = 1.000) and Lubombo and Shiselweni (*p* = 1.000) was not statistically significant.

The mean availability of LPGs was fairly high in Lubombo and Shiselweni and high in Manzini and Hhohho, with the highest availability of 83.3% recorded in Manzini, followed by 82.4% in the Hhohho region, then Shiselweni with 79.6% and lastly Lubombo region with an availability of 76.2%. The differences were not statistically different (*p* < 0.001).

The overall mean availability of OBs in the surveyed outlets varied from 0.0% for medicines like simvastatin and glibenclamide to 88.5% for metformin. The higher mean availability of the LPGs was evident with 30 out of the 50 surveyed medicines recording more than 90.0% availability and six medicines with 100.0% availability.

Results showed that the pharmacies located within cities had a higher OB mean availability (*x* = 54.3%, s.d. = 24.6%) as compared to the outlets situated in towns and rural areas (*x* = 28.6%, s.d. = 19.4%). The mean difference (25.7%) between the two locations was statistically significant (*p* = 0.001).

The availability of LPGs was high in the city and fairly high in the town located pharmacies, with pharmacies in the cities registering 87.4% availability and the outlets in towns and rural areas recording 76.5% availability. Results showed that this difference was statistically significant (*p* = 0.001).

The overall availability of all the different groups analysed was generally high for the LPGs and low for the OBs. The global core medicines had the highest LPG mean availability (89.2%; s.d. = 9.0%), followed by AI at 86.7%, then EML medicines with 85.1%, followed by 82.3% for the NCD medicines and then 77.7% overall mean availability for the supplementary medicines (Table 1). The differences between the different regions in terms of availability of LPGs of the different classes were not statistically significant. A similar trend was observed with respect to availability of the different medicines’ groups in both cities and smaller towns and the rural areas.

### Consolidated private retail sector patient price ratios

Of the 50 medicines surveyed in the 32 outlets, price ratios were calculated for 33 OBs and 48 LPGs (where medicines were found in four or more outlets). Overall, the MPR was 18.60 for the OBs, whilst the MPR for the LPGs for Eswatini retail outlets was 4.51. The 25th and 75th percentile MPRs for OBs were 3.91 and 36.75, respectively, with an MPR range of 132.58 (0.70–133.28), and for the LPGs, they were 1.98 and 10.36, respectively, with 0.23 as the minimum MPR and 60.72 as the maximum.

### Regional patient price comparison

Overall patient prices in Shiselweni were approximately 4% and 8% more than patient prices in Manzini region, whilst the Lubombo prices were approximately 42% and 9% more in comparison with Manzini for OBs and LPGs, respectively. The overall MPR patient prices in Shiselweni were approximately 7% cheaper than the prices in Hhohho for the LPGs. Analysis of variance results showed there was no significant difference between the regions in terms of LPG prices (*p* < 0.719).

A few of the LPGs had large price differentials across the different regions. The LPG omeprazole 20 mg cap/tab was sold to patients at 3.18 MPR in Manzini, but was available at more than four times that price in Lubombo and Shiselweni regions. Comparatively in the Hhohho region, the patients had to part with almost 10 times the Manzini price. The LPG furosemide was available at 2.12 MPR in Manzini, required more than 3 times this in Lubombo and Shiselweni, and was available at 9.38 MPR in the Hhohho region. Atenolol 50 mg, diclofenac 50 mg and fluoxetine 20 mg also showed large price differentials across the regions.

Overall patient prices in the cities were 3% more and 5% less than patient prices in the smaller towns for OBs and LPGs, respectively. The LPG diazepam 5 mg was available to patients at 1.12 MPR to patients in the cities, whilst it was sold at 6.58 MPR in the smaller towns.

OB price ratios were only analysed for Hhohho and Manzini regions as the other two regions did not have significant OB availability for any meaningful comparison. Overall, patient prices in Manzini were approximately 3% and 1% more than patient prices in the Hhohho region for OBs and LPGs, respectively. Results showed that the difference between Manzini and Hhohho OB medicines’ prices was not statistically significant (*p* = 0.166).

### Comparison of retail prescription drug prices in Eswatini and South Africa

Of the 32 OBs that were found in more than four outlets in Eswatini only two products, diclofenac 50 mg capsule/tablet (cap/tab) and amlodipine 5 mg cap/tab had their highest prices equal to the maximum permissible South African patient price, whilst eight products had their Eswatini highest prices lower than the maximum permissible South African patient prices, and the rest had Eswatini highest prices that were more than the maximum permissible South African patient prices (see Table 3). The differences were statistically significant (*p* = 0.004). Only one product, carvedilol 12.5 mg cap/tab, had its lowest Eswatini unit price lower than the cheapest South African patient price, whilst the lowest prices for all the other OBs were higher than the lowest prices in South Africa of the same products. The differences were statistically significant (*p* = 0.14).

Of the 48 LPGs analysed, one product, tamsulosin, had its Eswatini lowest price equal to South Africa’s lowest generic patient price, 26 products’ Eswatini lowest prices were higher than South Africa’s lowest prices and 18 products had their lowest prices lower than corresponding South African LPGs for the same molecules. The lowest prices of simvastatin 20 mg and omeprazole 20 mg were more than 300% lower than the lowest generic patient prices in South Africa. The lowest and highest LPG prices for South Africa were calculated using the MRPs from the Mediscor platform.^34^

Of all the LPGs that were analysed, only 11 products had Eswatini highest prices lower than the South African maximum permissible patient prices in the retail pharmacies. The other 37 products had highest prices, higher than the highest generic patient prices for similar molecules in South Africa. The difference in the prices was statistically significant (*p* = 0.014).

### Treatment affordability

The LPGs for the most common AI were generally affordable in all the regions, with an adult 7 days’ course of amoxicillin, a paediatric 7 days’ course of co-trimoxazole and a paediatric 7 days’ course of amoxicillin + clavulanic acid, all requiring less than a day’s wages of the lowest paid government unskilled worker to purchase them (see Table 4). The OB for amoxicillin and clavulanic acid, however, required 2.28 days’ wages in Manzini and 2.13 days’ wages in the Hhohho region. A single dose of 1 g azithromycin for the treatment of chlamydial infections was unaffordable for both LPGs and OBs in all the regions, save for Shiselweni region where purchasing the LPG course required only 0.94 days’ wages of the lowest paid unskilled government worker.

A standard 1-month treatment course for benign prostate hypertrophy (BPH) with tamsulosin 0.4 mg once daily was unaffordable in all the four regions for both the LPGs and OBs. The lowest paid unskilled government worker needed to work for 3 days to afford the LPG and 5.98 days to be able to buy the OB in Manzini region.

Management of depression with fluoxetine 20 mg once daily was affordable for the LPGs in all the regions requiring 0.56 days’ worth of wages of the lowest paid government unskilled worker Manzini, 0.88 days’ wages in Shiselweni, 0.95 days’ wages in Lubombo and 0.96 days’ wages in the Hhohho region (Table 2). The OB was only available in Hhohho and required the lowest paid unskilled government employee to work 7.16 days to afford to purchase a course for 1 month.

**TABLE 2:** Eswatini median price ratio comparison by location.

Medicine name (LPG)	Median price ratio (MPR)					
	Manzini (*n* = 12)	Hhohho (*n* = 8)	Shiselweni (*n* = 6)	Lubombo (*n* = 5)	Town/rural (*n* = 19)	City (*n* = 13)
Acyclovir 200 mg cap/tab	5.79	5.66	5.33	5.02	5.53	5.6
Amitriptyline 25 mg cap/tab	9.98	11.07	9.27	12.47	10.00	10.79
Amlodipine 5 mg cap/tab	11.68	11.68	10.29	13.99	11.69	11.67
Amoxicillin 500 mg cap/tab	2.26	2.10	2.63	2.58	2.49	2.01
Amoxicillin + clavulanic acid 125 mg + 31.25 mg susp	0.23	0.21	0.20	0.30	0.26	0.22
Anhydrous theophylline 200 mg cap/tab	3.85	-	3.19	3.73	3.63	3.85
Atenolol 50 mg cap/tab	8.00	6.71	12.08	4.20	7.88	8.11
Atorvastatin 20 mg cap/tab	0.97	0.92	0.99	1.37	1.09	0.87
Azithromycin 500 mg cap/tab	13.00	14.52	10.13	15.59	13.3	14.22
Beclomethasone 100 mcg/dose Inh	1.63	1.55	1.51	1.55	1.5	1.59
Bisoprolol 5 mg cap/tab	1.55	1.78	1.54	2.01	1.67	1.57
Budesonide 0.5 mg/mL Neb susp	59.44	62.40	-	-	61.27	60.72
Captopril 25 mg cap/tab	1.27	1.10	1.46	1.56	1.46	1.22
Carbamazepine 200 mg cap/tab	10.48	10.95	10.68	11.92	11.1	10.71
Carvedilol 12.5 mg cap/tab	1.31	1.31	1.31	1.43	1.32	1.31
Ceftriaxone injection 1 g/vial	6.49	5.78	8.17	8.17	8.17	5.77
Cetirizine 10 mg cap/tab	14.77	16.97	14.98	17.75	16.62	15.06
Ciprofloxacin 500 mg cap/tab	6.51	6.89	7.47	7.85	7.46	6.7
Co-trimoxazole suspension 8 mg + 40 mg/5 mL susp	2.12	1.90	2.36	2.39	2.3	1.87
Diazepam 5 mg cap/tab	2.22	1.00	-	-	6.58	1.12
Diclofenac 50 mg cap/tab	6.51	7.09	9.57	9.57	9.57	6.64
Enalapril 20 mg cap/tab	12.30	14.55	9.45	16.96	12.33	12.54
Fluoxetine 20 mg cap/tab	2.12	3.63	3.33	3.58	3.41	3.27
Fluticasone/salmeterol 25/250 mg Inh	10.16	11.01	-	10.80	10.8	11.01
Furosemide 40 mg cap/tab	2.12	9.38	6.59	7.69	6.59	4.33
Glibenclamide 5 mg cap/tab	4.69	4.60	4.14	6.05	4.47	4.47
Gliclazide 80 mg cap/tab	1.84	1.90	1.76	1.77	1.76	2.01
Hydrochlorothiazide 25 mg cap/tab	6.56	9.05	8.04	8.74	8.74	6.4
Hydrochlorothiazide/losartan 12.5/50 mg cap/tab	1.78	1.79	-	-	1.6	1.79
Irbesartan 150 mg cap/tab	10.40	10.61	9.92	11.04	10.83	10.21
Loratadine 10 mg cap/tab	6.20	7.13	5.97	6.53	6.15	7.09
Losartan 10 mg cap/tab	1.93	2.13	1.72	2.22	1.92	2.07
Metformin 500 mg cap/tab	2.55	2.41	2.25	2.22	2.39	2.34
Methyldopa 250 mg cap/tab	2.57	2.65	2.39	2.66	2.66	2.47
Montelukast 5 mg cap/tab	6.61	7.07	7.14	7.25	7.14	7.03
Nifedipine retard 20 mg cap/tab	4.80	4.64	4.41	5.19	4.55	4.57
Omeprazole 20 mg cap/tab	3.18	31.48	14.26	14.26	14.26	20.45
Paracetamol suspension 24 mg/mL susp	1.67	1.69	2.04	2.21	2.05	1.68
Phenytoin 100 mg cap/tab	4.94	4.99	5.20	5.84	5.52	4.98
Propranolol 40 mg cap/tab	3.57	3.02	3.45	4.16	3.83	3.42
Risperidone 2 mg cap/tab	57.91	62.09	50.40	-	54.41	60.59
Salbutamol inhaler 100 mcg/dose	1.57	1.58	1.35	1.65	1.46	1.55
Salbutamol syrup 2 mg/5 mL	2.40	2.55	2.46	2.63	2.58	2.51
Simvastatin 20 mg cap/tab	1.79	1.86	1.58	1.70	1.68	1.86
Sodium valproate 200 mg cap/tab	3.67	4.16	3.67	3.37	3.55	4.08
Spironolactone 25 mg cap/tab	3.06	3.22	2.84	2.98	2.98	3.11
Tamsulosin 0.4 mg cap/tab	10.60	11.13	10.72	10.81	10.76	10.72

mg, milligrams per dosage form; cap/tab, capsules or tablets as a dosage form; mcg/dose, micrograms per dose of medicine; Inh, inhaler; Neb susp, Nebulising suspension; LPG, lowest price generic.

**TABLE 3:** Comparison of Eswatini and South Africa’s highest and lowest originator brand prescription prices in Emalangeni (E).

Medicine name	Eswatini highest unit price (E)	S.A. maximum permissible unit price (E)	Price difference (%) Eswatini relative to S.A.	Lowest unit price – Eswatini (E)	S.A. lowest unit price (E)	% price difference
Salbutamol inhaler 100 mcg/dose	1.91	0.72	+62	0.51	0.41	+20
Sodium valproate 200 mg cap/tab	13.27	6.45	+51	5.72	4.69	+18
Hydrochlorothiazide/losartan 12.5/50 mg cap/tab	14.68	7.80	+47	6.09	4.85	+20
Human insulin 30/70 isophane 100 iu/mL vial	77.14	49.25	+36	41.11	33.88	+18
Risperidone 2 mg cap/tab	55.40	35.44	+36	35.94	27.52	+23
Carbamazepine 200 mg cap/tab	9.67	6.98	+28	6.66	4.58	+31
Bisoprolol 5 mg cap/tab	9.29	7.18	+23	5.12	4.40	+14
Fluticasone/salmeterol 25/250 mg Inh	17.51	15.27	+13	14.55	11.67	+20
Montelukast 5 mg cap/tab	25.99	23.06	+11	19.86	16.78	+16
Omeprazole 20 mg cap/tab	36.37	32.77	+10	31.24	25.05	+20
Fluoxetine 20 mg cap/tab	21.73	19.61	+10	17.53	14.02	+20
Metformin 500 mg cap/tab	1.49	1.36	+9	0.86	0.70	+19
Phenytoin 100 mg cap/tab	4.82	4.44	+8	3.61	2.97	+18
Spironolactone 25 mg cap/tab	2.84	2.63	+7	1.84	1.52	+17
Tamsulosin 0.4 mg cap/tab	18.78	17.38	+7	14.01	12.12	+13
Atorvastatin 20 mg cap/tab	21.20	19.77	+7	15.25	14.15	+7
Irbesartan 150 mg cap/tab	18.47	17.51	+5	12.55	11.92	+5
Ciprofloxacin 500 mg cap/tab	33.00	32.41	+2	24.73	21.07	+15
Losartan 10 mg cap/tab	7.11	7.02	+1	5.02	4.28	+15
Budesonide 0.5 mg/mL Neb susp	28.77	28.48	+1	24.63	20.23	+18
Enalapril 20 mg cap/tab	6.78	6.72	+1	4.87	3.97	+18
Diclofenac 50 mg cap/tab	6.06	6.04	0	4.08	3.35	+18
Amlodipine 5 mg cap/tab	11.28	11.30	0	9.25	7.38	+20
Furosemide 40 mg cap/tab	9.03	9.16	-1	6.65	4.87	+27
Azithromycin 500 mg cap/tab	107.29	109.53	-2	85.58	71.32	+17
Atenolol 50 mg cap/tab	13.21	13.53	-2	11.25	9.00	+20
Nifedipine retard 20 mg cap/tab	14.82	15.54	-5	14.28	11.90	+17
Loratadine 10 mg cap/tab	5.98	6.69	-12	2.61	2.00	+23
Carvedilol 12.5 mg cap/tab	9.15	10.50	-15	6.66	6.80	-2
Amoxicillin + clavulanic acid 125 mg + 31.25 mg susp	2.05	2.38	-16	1.66	1.48	+11
Cetirizine 10 mg cap/tab	9.19	10.80	-18	8.59	7.02	+18

Note: $1.00 is equivalent to 14.79 Swazi lilangeni (SZL).

mg, milligrams per dosage form; cap/tab, capsules or tablets as a dosage form; mcg/dose, micrograms per dose of medicine; S.A., South Africa; Inh, inhaler; iu, international unit; E, Emalangeni; Neb susp, Nebulising suspension.

**TABLE 4:** Affordability of selected lowest price generic medicines.

Medicine	Number of days’ wages needed to purchase a course of treatment			
	Manzini	Hhohho	Shiselweni	Lubombo
Amoxicillin 500 mg cap/tab	0.29	0.27	0.34	0.33
Azithromycin 500 mg cap/tab	1.20	1.35	0.94	1.45
Beclomethasone 100 mcg/dose Inh	0.84	0.80	0.78	0.80
Carbamazepine 200 mg cap/tab	2.38	2.48	2.42	2.70
Enalapril 20 mg cap/tab	0.86	1.02	0.66	1.19
Fluoxetine 20 mg cap/tab	0.56	0.96	0.88	0.95
Fluticasone/salmeterol 25/250 mg/dose Inh	5.94	6.44	-	6.32
Glibenclamide 5 mg cap/tab	0.33	0.32	0.29	0.42
Gliclazide 80 mg cap/tab	1.10	1.14	1.05	1.06
Human insulin 100 iu/mL Inj	5.20	5.36	-	7.37
Hydrochlorothiazide 25 mg cap/tab	0.17	0.24	0.21	0.23
Irbesartan 150 mg cap/tab	1.89	1.93	1.80	2.00
Losartan 50 mg cap/tab	1.36	1.50	1.22	1.57
Metformin 500 mg cap/tab	0.47	0.44	0.41	0.41
Methyldopa 500 mg cap/tab	1.53	1.58	1.42	1.59
Montelukast 5 mg cap/tab	3.40	3.63	3.67	3.73
Nifedipine SR 20 mg cap/tab	1.20	1.16	1.10	1.29
Omeprazole 20 mg cap/tab	0.74	2.72	1.23	1.23
Salbutamol 100 mcg/dose Inh	0.18	0.18	0.16	0.19
Sodium valproate 200 mg cap/tab	3.13	3.55	3.13	2.87
Tamsulosin 0.4 mg cap/tab	2.93	3.08	2.97	2.99

mg, milligrams per dosage form; cap/tab, capsules or tablets as a dosage form; mcg/dose, micrograms per dose of medicine; Inh, inhaler; iu, international units; SR, sustained release.

The first-line LPG anti-hypertensives (hydrochlorothiazide, captopril and atenolol) were affordable in all the four regions of Eswatini requiring less than a day’s wage to purchase a course, with hydrochlorothiazide being the most affordable, requiring only under 0.25 day’s wages of the lowest paid unskilled government employee to purchase it. The second-line and third-line anti-hypertensives were generally unaffordable, requiring more than a day’s wages for both the OBs and LPGs, apart from the LPGs for 20 mg enalapril in Manzini (0.86 days) and Shiselweni (0.66 days).

The three anti-epileptic medicines surveyed required more than a day’s wages to buy a course for 1 month, save for the LPGs for phenytoin 100 mg tablets which required 0.94 days’ wages in Manzini and 0.95 days’ wages in the Hhohho region. To afford a course of sodium valproate for a month, the lowest paid government worker had to work for 4.52 days for the OB and 3.55 days for the LPG. A similar trend was observed with carbamazepine as well, with the most affordable OB requiring 4.19 days’ wages, whilst the most affordable LPG required 2.38 days’ wages to purchase a course.

A course of salbutamol inhaler required less than 0.2 day’s wages for the LPGs and just under 0.5 days’ wages for the OB in all the regions where stock was available. Less than a single day’s wages were adequate to purchase a month’s supply of the LPGs of beclomethasone 100 mg inhaler in all the regions surveyed. Management of asthma with montelukast 5 mg once daily was unaffordable for both LPGs and OBs (requiring more than 3 days’ wages for the LPGs and just under 8 days’ wages for the OBs), as was fluticasone/salmeterol 25/250 mcg which required about 6 days’ wages for the LPGs and more than 10 days’ wages to purchase a monthly course of the OBs.

The first line of management for diabetes mellitus 2 (DM II) was affordable for both the OBs and LPGs in all the four regions of Eswatini, with a monthly course of 60 tablets of glibenclamide 5 mg requiring less than 0.5 days’ wages to purchase. It is worth noting that less than 0.8 days’ wages were required to purchase a course of either the OBs or the LPGs of metformin 500 mg in the four regions. Gliclazide was less affordable and required more than the equivalent of the lowest paid unskilled government worker’s single day wage to purchase a course for 1 month of the LPGs. No LPG was found for human insulin (30% regular/70% isophane) in all the surveyed areas and the OB required 5.20 days’ wages in Manzini, 5.36 days’ wages in Hhohho and 7.37 days’ wages in the Lubombo region.

Table 5 illustrates the affordability of a 3-drug regimen when OBs and LPGs when purchased from the retail pharmacy sector in Eswatini. The lowest paid government worker would have to work 2 days to afford the LPG regimen, and in case the LPGs are not available, she/he will have to work for 4.8 days to be able to afford the OBs.

**TABLE 5:** Affordability of treatments for a family with multiple conditions (consolidated retail pharmacy sector—Eswatini).

Condition	Treatment	Type	Median treatment price (emalangeni)	Days’ wages
Hypertension	Enalapril 20 mg od × 30 days	LPG	74.66	0.9
		OB	164.93	1.9
Diabetes mellitus II	Metformin 500 mg twice daily × 30 days	LPG	36.60	0.4
		OB	60.47	0.7
Respiratory infection (child)	Amoxicillin + clavulanic acid 125/31.25 mg 3 times daily × 7 days	LPG	60.40	0.7
		OB	187.58	2.2

**Total**	**-**	**LPG**	**171.66**	**2.0**
		**OB**	**412.98**	**4.8**

OB, originator brand; LPG, lowest priced generic equivalent; Od, once a day.

## Discussion

The study showed that the overall availability of the LPG medicines in all the regions was higher than the recommended minimum availability benchmark of 80%^20^ set by WHO/HAI. It can be deduced from the results that there is promotion of generics’ dispensing as compared to the branded products in all the regions. The more affluent settlements are in Hhohho and Manzini regions, and the availability of OBs was comparably higher in these two regions. Guan et al.’s^35^ findings highlighted the challenges associated with regional disparity of essential medicines. Another study looked at the undiscounted prices for both OB and the LPG for 25 essential medicines from 17 private pharmacies in Shaanxi Province, western China. It noted that generics were more available as compared to OBs and prices varied across different discount programmes. The study concluded that price transparency of pharmaceuticals helps consumers in the identification of potential savings.^36^

The overall availability of LPGs was not different in all the regions. However, the pharmacies located in cities had higher availability than the ones in smaller towns and rural areas for both LPGs and OBs. A survey conducted in Peru using the WHO/HAI medicines’ prices and availability survey did not find any significant differences in overall availability or prices of the medicines under study by retail location.^15^ It is commendable that the availability of LPGs on the EML was more than 90% in all regions, and as such in the event of medicines being unavailable at government hospitals, patients can access medications at retail pharmacies.

It was quite interesting to note that the LPGs’ prices were comparable in all the four regions and the differences reflected were not statistically significant. Although there is currently no price regulation administered by the government,^37^ the market forces ‘regulate’ the medicines’ prices in the Eswatini pharmaceutical market. Studies on medicines’ prices, availability and affordability carried out in Nairobi County in 2016^38^ and in Sudan from March 2012 to April 2013 noted considerable price differences amongst the different regions despite having people of similar social standing.^22^

A careful analysis of the molecules that had large differentials, for example, omeprazole 20 mg, showed that the overseas parallel generic imports were available at a significantly lower price to the patients as compared to similar generic molecules that were sourced regionally. The clients in the more affluent locations are generally brand-sensitive as compared to patients in the rural settings, where outlets can afford to keep the most affordable non-branded import generics. There is a need for the authorities to ascertain that these lowly priced molecules also meet the stipulated quality standards.

The study showed that generally the OB patient prices were higher in Eswatini when compared to prices of the same prescription molecules in South Africa, and this is not a deviation from the expected as literature suggests medicines’ prices in regulated environments are generally lower than in countries with no price regulation.^23^ Eswatini procures their OBs from South Africa where pricing is regulated, and as such, the best/lowest cost to the retail outlets for these would be at the SEP price and as such it is expected that the retailing prices will be higher than SEP in the Eswatini outlets. Eswatini prescription medicines’ prices have VAT at 0%,^4^ whereas in South Africa all medicines attract VAT at 15%. If the VAT regulation in Eswatini would change from 0% to the standard 15%,^24^ the Eswatini prescription medicines will become more expensive to the end users.

The Eswatini market is not limited to the South African generic molecules only as they also have access to parallel import generics from overseas which were found to be generally cheaper than the South African equivalent molecules. The difference in the gross domestic product (GDP) per capita per month between South Africa and Eswatini needs to be considered,^8^ as a small price difference may turn out to be huge with respect to affordability in the Eswatini context.

Most first-line treatment regimens for NCDs were generally affordable, requiring less than a day’s wages. It is worth noting that more than 60% of Eswatini’s population lives below the upper poverty line of $8.21 per capita per month, and as such,^8^ treatment regimens calculated as affordable may still be way out of the range for the general populace.^16^ There is usually more than one family member requiring chronic medications, and even though the individual courses may be affordable, the combined regimens will be unaffordable. Anti-epileptic medications and most second-line management regimes required more than a day’s wages; hence, unavailability at the public hospitals could lead to patients defaulting their treatments.

### Limitations

Affordability was calculated based on the daily wage of the lowest paid unskilled government worker, but a large portion of the labour force is not employed by the government and the minimum wages are way lower than the salary of the lowest paid government worker, thus the data may not be a true reflection of affordability in Eswatini.

Availability refers to the day of data collection at each facility and might not be indicative of average availability over time. Availability and prices’ data were collected during level 2 lockdown (as a result of the [coronavirus disease 2019] COVID-19 pandemic) when supply chains were disrupted, and hence, these findings may not be a true reflection of the availability throughout the year (Eswatini depends entirely on imports as there is no manufacturing of any pharmaceuticals that takes place in the kingdom).

### Recommendations

Future research should focus on comprehensive national surveys in all public and private entities to determine medicines’ prices, including from the wholesalers. Price components throughout the entire pharmaceutical supply chain should be studied. Focus on these will assist in developing policies that will work towards improving affordability and availability.

## Conclusion

Drug pricing control by the government is one of the factors responsible for lower retail prices in South Africa. The concept of ‘free market economy’ in Eswatini may not be enough to regulate the prices of medicines. There is a need to develop drug pricing policies that govern the whole supply chain.^16^ However, for that to happen all the necessary data on the current pricing structure in the whole pharmaceutical supply chain of Eswatini should be gathered. The *Medicines and Related Substances Act of 2016* allows for the implementation of a pricing system for medicines in Eswatini.^17^

## Appendix 1

**TABLE 1-A1:** Medicines’ classification.

Medicine name (including strength and dosage form)	WHO/HAI list	Anti-infective/NCD	EML
Acyclovir 200 mg cap/tab	S	AI	EML
Amitriptyline 25 mg cap/tab	G	NCD	EML
Amlodipine 5 mg cap/tab	S	NCD	EML
Amoxicillin 500 mg cap/tab	G	AI	EML
Amoxicillin + clavulanic acid 125 mg + 31.25 mg susp	S	AI	EML
Anhydrous theophylline 200 mg cap/tab	S	NCD	EML
Artemether/lumefantrine 20 mg/120 mg	S	AI	EML
Atenolol 50 mg cap/tab	S	NCD	EML
Atorvastatin 20 mg cap/tab	S	NCD	EML
Azithromycin 500 mg cap/tab	S	AI	EML
Beclomethasone 100 mcg/dose Inh	S	NCD	EML
Bisoprolol 5 mg cap/tab	G	NCD	EML
Budesonide 0.5 mg/mL Neb susp	S	NCD	NEML
Captopril 25 mg cap/tab	G	NCD	EML
Carbamazepine 200 mg cap/tab	S	NCD	EML
Carvedilol 12.5 mg cap/tab	S	NCD	EML
Ceftriaxone injection 1 g/vial	G	AI	EML
Cetirizine 10 mg cap/tab	S	NCD	EML
Ciprofloxacin 500 mg cap/tab	G	AI	EML
Co-trimoxazole suspension 8 mg + 40 mg/5 mL susp.	G	AI	EML
Diazepam 5 mg cap/tab	G	NCD	EML
Diclofenac 50 mg cap/tab	G	NCD	EML
Enalapril 20 mg cap/tab	S	NCD	EML
Fluoxetine 20 mg cap/tab	S	NCD	EML
Fluticasone/Salmeterol 25/250 mg Inh	S	NCD	NEML
Furosemide 40 mg cap/tab	S	NCD	EML
Glibenclamide 5 mg cap/tab	S	NCD	EML
Gliclazide 80 mg cap/tab	S	NCD	EML
Hydrochlorothiazide 25 mg cap/tab	S	NCD	EML
Hydrochlorothiazide/losartan 12.5/50 mg cap/tab	S	NCD	NEML
Irbesartan 150 mg cap/tab	S	NCD	EML
Loratadine 10 mg cap/tab	S	NCD	NEML
Losartan 10 mg cap/tab	S	NCD	NEML
Metformin 500 mg cap/tab	G	NCD	EML
Methyldopa 250 mg cap/tab	S	NCD	EML
Montelukast 5 mg cap/tab	S	NCD	NEML
Nifedipine retard 20 mg cap/tab	S	NCD	EML
Omeprazole 20 mg cap/tab	G	NCD	EML
Paracetamol suspension 24 mg/mL susp	G	NCD	EML
Phenytoin 100 mg cap/tab	S	NCD	EML
Propranolol 40 mg cap/tab	S	NCD	EML
Risperidone 2 mg cap/tab	S	NCD	NEML
Salbutamol inhaler 100 mcg/dose	G	NCD	EML
Salbutamol syrup 2 mg/5 mL	S	NCD	EML
Simvastatin 20 mg cap/tab	G	NCD	EML
Sodium valproate 200 mg cap/tab	S	NCD	EML
Spironolactone 25 mg cap/tab	S	NCD	EML
Tamsulosin 0.4 mg cap/tab	S	NCD	NEML

mg, milligrams per dosage form; cap/tab, capsules or tablets as a dosage form; mcg/dose, micrograms per dose of medicine; S, supplementary list; G, Global Core List; NCD, non-communicable disease; AI, anti-infectives; EML, essential medicines list; NEML, not on essential medicines list; Inh, inhaler; Neb susp, Nebulising suspension; WHO/HAI, World Health Organization/Health Action International.
